# Coordination of {Mo_142_} Ring to La^3+^ Provides Elliptical {Mo_134_La_10_} Ring with a Variety of Coordination Modes

**DOI:** 10.3390/ma3010064

**Published:** 2009-12-28

**Authors:** Eri Ishikawa, Yutaka Yano, Toshihiro Yamase

**Affiliations:** 1 Chemical Resources Laboratory, Tokyo Institute of Technology, R1-21, 4259 Nagatsuta, Midori-ku, Yokohama 226–850, Japan; 2 Department of Applied Chemistry, College of Engineering, Chubu University, 1200 Matsumoto, Kasugai, Aichi 487–8501, Japan; E-Mail: eishikawa@isc.chubu.ac.jp (E.I.); 3 Matsuda Sangyo Co., Ltd., Shinjuku Nomura Building 6th Fl., 1-26-2 Nishishinjuku, Shinjuku-ku, Tokyo 163–0558, Japan; E-Mail: yano-y@matsuda-sangyo.co.jp (Y.Y.); 4 (Inc.) MO Device, 2-14-10 Kanaiwa-higashi, Kanazawa 920–0335, Japan

**Keywords:** {Mo_142_}- ring, elliptical {Mo_134_La_10_}-ring, Mo-blues molecular design, La^3+^-coordination geometry, X-ray crystal structure, isothermal titration calorimetry (ITC), ^139^La-NMR

## Abstract

A 28-electron reduced *C_2h_*-Mo-blue 34Ǻ outer ring diameter circular ring, [Mo_142_O_429_H_10_(H_2_O)_49_(CH_3_CO_2_)_5_(C_2_H_5_CO_2_)]^30-^ (≡{Mo_142_(CH_3_CO_2_)_5_(C_2_H_5_CO_2_)}) comprising eight carboxylate-coordinated (with disorder) {Mo_2_} *linkers* and six defect pockets in two inner rings (four and three for each, respectively), reacts with La^3+^ in aqueous solutions at pH 3.5 to yield a 28-electron reduced elliptical *C_i_-*Mo-blue ring of formula [Mo_134_O_416_H_20_(H_2_O)_46_{La(H_2_O)_5_}_4_{La(H_2_O)_7_}_4_{LaCl_2_(H_2_O)_5_}_2_]^10-^ (≡{Mo_134_La_10_}), isolated as the Na_10_[Mo_134_O_416_H_20_(H_2_O)_46_{La(H_2_O)_5_}_4_{La(H_2_O)_7_}_4_{LaCl_2_(H_2_O)_5_}_2_]·144 H_2_O Na^+^ salt. The elliptical structure of {Mo_134_La_10_} showing 36 and 31 Å long and short axes for the outer ring diameters is attributed to four (A-D) modes of LaO_9_/LaO_7_Cl_2 _tricapped-trigonal-prismatic coordination (TTP) geometries. Two different LaO_2_(H_2_O)_7_ and one LaO_2_(H_2_O)_2_Cl_2_ TTP geometries (as A-C modes) for each of two inner rings result from the coordination of all three defect pockets of the inner ring for {Mo_142_(CH_3_CO_2_)_5_(C_2_H_5_CO_2_)}, and two LaO_4_(H_2_O)_5_ TTP geometries (as D mode) result from the displacement of two (acetate/propionate-coordinated) binuclear {Mo_2_} *linkers* with La^3+^ in each inner ring. The isothermal titration calorimetry (ITC) of the ring modification from circle to ellipsoid, showing the endothermic reaction of [La^3+^]/[{Mo_142_(CH_3_CO_2_)_5_(C_2_H_5_CO_2_)}] = 6/1 with *ΔH* = 22 kJ⋅mol^-1^, *ΔS* = 172 J⋅K^-1^⋅mol^-1^, *ΔG* = −28 kJ⋅mol^-1^, and *K* = 9.9 × 10^4^ M^-1^ at 293 K, leads to the conclusion that the coordination of the defect pockets to La^3+^ precedes the replacement of the {Mo_2_} *linkers* with La^3+^. ^139^La- NMR spectrometry of the coordination of {Mo_142_(CH_3_CO_2_)_5_(C_2_H_5_CO_2_)} ring to La^3+^ is also discussed.

## 1. Introduction

**Figure 1:**
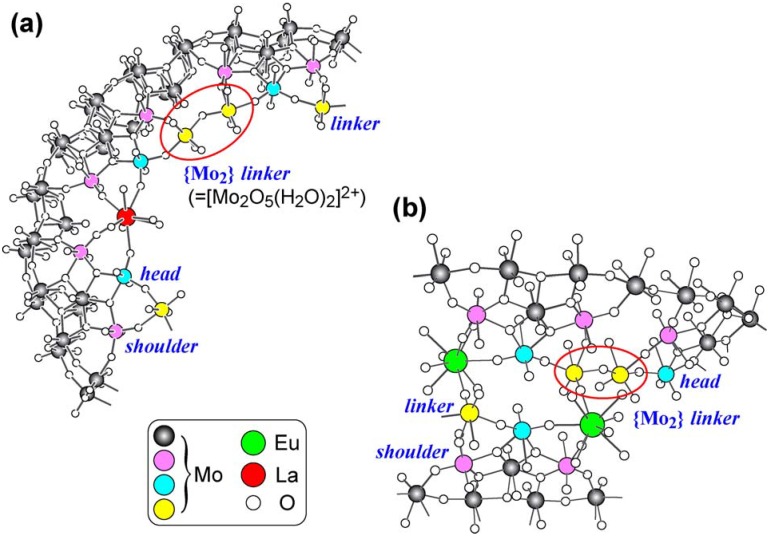
Environments of the La-coordination [2] in {Mo_150_La_2_} (a) and the Eu-coordination [3] in {Mo_128_Eu_4_}_2_ (b). Pink-, blue- and yellow colored Mo atoms are at *shoulder*, *head* and *linker* Mo sites, respectively.

The Mo^VI^→Mo^V^ reduction of isopolyoxomolybdates in strongly acidic aqueous solutions containing trivalent lanthanide cations (Ln^3+^) provides for the incorporation of Ln^3+^ into the Mo-blue ring circles with a resultant modification of the ring shape to a Japanese rice-ball shape: [Mo_120_{Pr(H_2_O)_5_}_6_O_366_H_12_(H_2_O)_48_]^6−^ (≡{Mo_120_Pr_6_}) [1] and [Mo_120_O_366_H_14_(H_2_O)_48_{La(H_2_O)_5_}_6_]^4−^ (≡{Mo_120_La_6_}) [2] have been prepared by the hydrazinium-dichloride reduction of the Na_2_MoO_4_/ Pr(NO_3_)_3_/HCl system at 70 °C and the UV-photolysis of the [NH_4_]_6_[Mo_7_O_24_]/LaCl_3_/*p*-CH_3_C_6_H_4_SO_2_Na system at pH 1.2, respectively, and both complexes have been successfully characterized as Japanese rice-ball structured 24-electron reduced Mo-blues [1,2]. In addition, the species [Mo_150_O_452_H_2_(H_2_O)_66_{La(H_2_O)_5_}_2_]^24−^ (≡{Mo_150_La_2_}) [2] and [{Mo_128_Eu_4_O_388_H_10_(H_2_O)_81_}_2_]^20−^ (≡{Mo_128_Eu_4_}_2_) [3] prepared by the UV-photolysis of [NH_4_]_8_[Mo_36_O_112_(H_2_O)_16_]/LaCl_3_/[*^i^*PrNH_3_][ClO_4_] system at pH 1.0 and the hydrazinium-dichloride reduction of the K_2_MoO_4_/EuCl_3_/HCl system at 60–65 °C have been also characterized as 28- and 24-electron reduced elliptical Mo-blue rings, respectively. As well as the above Japanese rice-ball-shaped Mo-blues, the former has shown that the coordination of Ln^3+^ within the inner ring is the same as the one of the binuclear {Mo_2_} *linker* which coordinates four O atoms belonging to MoO_6_ octahedra of two *head* and two *shoulder* Mo sites (as a 4-*fold* ligand), as shown in Figure 1a [4,5,6,7]. Such a coordination of two sets of *head* and *shoulder* MoO_6_-octahedral sites to Ln^3+^, yielding the LnO_4_(H_2_O)_5_ tricapped-trigonal-prismatic coordination (TTP) geometry, arises from a strong electrostatic interaction with the negatively charged inner-ring compared with the {Mo_2_} ( = [Mo_2_O_5_(H_2_O)_2_]^2+^) *linker* to lead to the elliptical structure due to a larger molecular curvature, if we considered that Ln^3+^ is smaller in size than the {Mo_2_} *linker* [2]. On the other hand, the latter is a dimer of two elliptical rings (due to the insertion of two [Mo_2_O_7_(H_2_O)]^2-^ into the outer ring) linked by Eu^3+^ through two Mo-O-Eu-O-Mo bonds: four Eu atoms in the elliptical ring coordinate two O atoms belonging to *head* and *shoulder* MoO_6_ octahedra, and one of them is also bound to the O atom belonging to the MoO_6_ octahedron of the binuclear {Mo_2_} *linker* of the neighboring ring with a resultant EuO_3_(H_2_O)_6_ TTP geometry (Figure 1b), and other Eu atoms are in the EuO_2_(H_2_O)_7_ TTP geometry without coordination of the O atom belonging to the neighboring ring [3].

The presence of Ln^3+^ in the self-assembly process to the Mo-blue rings around at pH = 1 not only provides for the incorporation of Ln^3+^ into the inner ring, but also generates the Mo-blue ring-aggregates resulting from the dehydrated condensation between the two inner rings of neighboring Mo-blue rings, as exemplified by compounds like {[Mo_154_O_458_H_12_(H_2_O)_66_]^8-^}_∞_ (nano-tube) [8] and {[Mo_146_O_442_H_6_(H_2_O)_60_{La(H_2_O)}_2_}]^8-^}_∞_ (nano-chain) [9]. In our continuing work on the molecular design of the Mo-blue nano-rings by a use of Ln^3+^, the reaction of the Mo-blue rings with Ln^3+^ has been investigated in order to discuss the competitive coordination to Ln^3+^ between the displacement of the {Mo_2_} *linker* and the defect pocket within the inner ring, which is expected for 28-electron reduced defect ring, [Mo_142_O_429_H_10_(H_2_O)_49_(CH_3_CO_2_)_5_(C_2_H_5_CO_2_)]^30−^ (≡{Mo_142_(CH_3_CO_2_)_5_(C_2_H_5_CO_2_)}), comprising eight carboxylate-coordinated (with disorder) {Mo_2_} *linkers* ( = {Mo_2_(carboxylate)}) and six defect pockets in two inner rings (four and three for each, respectively) [10]. In this work we describe that the coordination of{Mo_142_(CH_3_CO_2_)_5_(C_2_H_5_CO_2_)} to La^3+^ at pH 3.5 provides a novel elliptical Mo-blue ring [Mo_134_O_416_H_20_(H_2_O)_46_{La(H_2_O)_5_}_4_{La(H_2_O)_7_}_4_{LaCl_2_(H_2_O)_5_}_2_]^10-^ (≡{Mo_134_La_10_}, with *C_i_* symmetry, isolated as the corresponding Na^+^ salt, Na_10_[Mo_134_O_416_H_20_(H_2_O)_46_{La(H_2_O)_5_}_4_{La(H_2_O)_7_}_4_{LaCl_2_(H_2_O)_5_}_2_]·144H_2_O, and discuss that the coordination of the defect pockets to La^3+^ precedes the displacement of {Mo_2_} *linkers* within the inner ring, with a help of the isothermal titration calorimetry (ITC) and ^139^La-NMR spectrometry. The obtained results give a good method for the modification of the ring shape from circle to ellipsoid.

## 2. Experimental Section

### 2.1. Synthesis of Na_10_[Mo_134_O_416_H_20_(H_2_O)_46_{La(H_2_O)_5_}_4_{La(H_2_O)_7_}_4_{LaCl_2_(H_2_O)_5_}_2_]·144H_2_O

[NH_4_]_27_[(CH_3_)_3_NH]_3_[Mo_142_O_429_H_10_(H_2_O)_49_(CH_3_CO_2_)_5_(C_2_H_5_CO_2_)]·150 ± 10H_2_O was synthesized according to the published procedure [10]. The precursor [NH_4_]_27_[(CH_3_)_3_NH]_3_[Mo_142_O_429_H_10_(H_2_O)_49_(CH_3_CO_2_)_5_(C_2_H_5_CO_2_)]·150 ± 10H_2_O (10.5 mg, 0.4 μmol) was added to a solution containing NaCl (29.7 mg, 510 μmol) and LaCl_3_·7H_2_O (5.7 mg, 15 μmol) in 15 mL of H_2_O. The resulting rhombohedral dark−blue colored crystals of Na_10_[Mo_134_O_416_H_20_(H_2_O)_46_{La(H_2_O)_5_}_4_{La(H_2_O)_7_}_4_{LaCl_2_(H_2_O)_5_}_2_]·144H_2_O were precipitated at 4 °C within two weeks with a yield of 4.5 mg (40 % based on Mo). Calcd.: Na, 0.90; La, 5.46; Mo, 50.53; Cl, 0.56; and H, 2.04 %. Found: Na, 0.87; La, 5.09; Mo, 50.82; Cl, 0.52; and H 1.93 %. IR (KBr pellet): ν(cm^−1^) = 1,622{m, δ(H_2_O)}, 965 (m), 903 (w), 872 (w), 634 (s), 561 (s). Manganometric redox titration showed the presence of 28 ± 1 Mo^V^ centers in {Mo_134_La_10_}. TG analysis performed on a Rigaku Thermoflex TG-DGC instrument, showing almost monotonous decrease up to 200 °C, indicated that the crystal water content per molecule was approximately 151, when the gravimetric loss observed at 200 °C was assumed to be due to the removal of all the aqua ligands and crystal water molecules. The estimated number of the crystal water molecules is close to the one (144) based on the X-ray crystal structure analysis.

### 2.2. Crystal Data for Na_10_[Mo_134_O_416_H_20_(H_2_O)_46_{La(H_2_O)_5_}_4_{La(H_2_O)_7_}_4_{LaCl_2_(H_2_O)_5_}_2_]·144H_2_O

Na_10_Mo_134_La_10_O_664_H_516_Cl_4_, *M* = 25760.40 gmol^−1^, space group P2_1_/c (No.14), a = 35.530(16), b = 37.05(2), c = 36.11(2) Å, *β* = 119.85(2) °, *V* = 41227(38) Å^3^, *Z* = 2, *ρ* = 2.075 gcm^−3^, *μ* = 25.74 cm^−1^, F(000) = 24408. Crystal size = 0.20 × 0.15 × 0.10 mm. Transmission factors = 0.51–0.77. Crystal was coated with paraffin oil and mounted in a loop. The intensity data were collected on a Rigaku RAXIS−RAPID imaging plate diffractometer with a graphite monochromatized MoK_α_ (λ = 0.71069 Å) radiation at −100 °C. Lorentz polarization effects and numerical absorption corrections (using the program Numabs and Shape, T. Higashi, *Program for absorption correction*, Rigaku Corporation, Tokyo, 1999) were applied to the intensity data, and H atoms were not included in the calculation. A total of 388109 reflections (ω scan and 2θ_max_ = 55.0 °) were collected of which 94422 unique reflections (R_int_ = 0.102) were used. All of the calculations were performed using the CrystalStructure software package (*CrystalStructure 3.8*: *Crystal Structure Analysis Package*, Rigaku and Rigaku/MSC 2000−2003). The structure was solved by direct methods (SHELXS−97) and refined by SHELXL−97 (1915 parameters) to *R*_1_ = 0.092 for 52411 reflections with *I* > 2σ(*I*), and *R* = 0.161 and *R*_w_ = 0.283 for all data. Anisotropic temperature factors were applied to all La, Na and Mo atoms except four Mo atoms (Mo57, Mo61, Mo62, and Mo67). All O, Cl, and the four Mo atoms were refined isotropically. The positional parameters of the four Mo atoms (Mo57, 61, 62, and 67) and ten O atoms (O72–75, O84–90, O102–104) were not refined. The thermal parameters of Mo57, Mo67, O72–75, and O102–104 were kept to be unchanged throughout a structure refinement. The thermal parameters of Mo61, Mo62, and O84–90 were also unchanged. The maximum and minimum residual electron densities were 8.97 and −6.13 eÅ^−3^, respectively. Seven large reflection peaks remained around four Mo atoms: 8.97 eÅ^−3^ at the distance of 2.33 Å from Mo57, 7.87 and 7.60 eÅ^−3^ at 0.89 and 0.83 Å respectively from Mo61, 7.79 and 7.29 eÅ^−3^ at 0.79 and 0.67 Å respectively from Mo62, and 7.25 and 6.31 eÅ^−3^ at 0.79 and 0.67 Å respectively from Mo67. The calculation of the bond valence sums (∑*s*) for all the oxygen atoms for La−O and Mo−O bonds [11] indicated the coordination of 104 aqua ligands (for ∑*s* < 0.4) to the anion. Further details of the crystal structure investigation may be obtained from the Fachinformationszentrum Karlsruhe, 76344 Eggenstein-Leopoldshafen Germany (Fax: (+49)7247-808-666; E-Mail: crysdata@fiz-karlsruhe.de, http://www.fiz-karlsruhe.de/request for deposited data.html), on quoting the depository number CSD-421263.

### 2.3. Isothermal Titration Calorimetry (ITC)

Isothermal calorimetric titration of the aqueous solution containing {Mo_142_(CH_3_CO_2_)_5_(C_2_H_5_CO_2_)} by LaCl_3_ was performed with an Omega isothermal titration calorimeter (MicroCal, Northampton, MA, USA). The solutions were prepared with 0.05 ionic-strength of NaCl in deionized distilled water. The aqueous solutions containing 0.02 mM {Mo_142_(CH_3_CO_2_)_5_(C_2_H_5_CO_2_)} was placed in the sample cell, and the reference cell was filled with ultra-pure water. To avoid the generation of the heat of neutralization, the pH level of the LaCl_3_ solution was adjusted to pH 3.5 (natural pH level of the {Mo_142_(CH_3_CO_2_)_5_(C_2_H_5_CO_2_)} solution) by adding HCl. Aliquots of the LaCl_3_ solution (10 μL, 3 mM) were injected into the sample cell at 3.5-min intervals. The titration curves were analyzed with a help of Origin software (ver. 5.0) provided by MicroCal. Experimental data were fitted to theoretical curves for determining the binding enthalpy per mole of LaCl_3_ (*ΔH*), the binding constant (*K*), and the number of binding La^3+^ ions per {Mo_142_(CH_3_CO_2_)_5_(C_2_H_5_CO_2_)} ring (*n*) as the reaction stoichiometry. In order to determine the molar enthalpy for the reaction of La^3+^ with the Mo-blue ring, the heat of dilution of La^3+^, which was easily estimated by adding the aqueous solution of LaCl_3_ into the aqueous solution of 0.05 M NaCl adjusted to pH 3.5, was subtracted from the experimental value on each addition. The goodness of the curve fitting is evaluated by the minimization (<10,000) of χ^2^, which is simply expressed as

χ^2^ = (1/*n^eff^*−*p*) ∑ [*y_i_*−*f*(*x_i_*; *p_1_*, *p_2_*,…)]^2^

where *n^eff^* = the total number of experimental points used in the fitting, *p* = total number of adjustable parameters, *y_i_* = experimental data points, and *f*(*x_i_*; *p_1_*, *p_2_*,…) = fitting function [12]. The best fit for each titration curve was determined based on χ^2^ and error values: χ^2^ = 871 for the titration curve at 278 K, χ^2^ = 1650 for 293 K, χ^2^ = 3270 for 313 K, and χ^2^ = 6549 for 333 K.

### 2.4. NMR Measurements

^139^La-NMR spectra were measured at 298 ± 1 K using Φ = 10-mm internal diameter NMR tubes on a JEOL AL-300 spectrometer at 42.5 MHz with 90° pulses. A line-broadening factor of 1 Hz was applied before FT. Chemical shifts were referenced to an external LaCl_3_ solution (1 M) for which the resonance was taken as δ = 0 ppm.

## 3. Results and Discussion

### 3.1. Crystal Structure of {Mo_134_(La)_10_}

**Figure 2:**
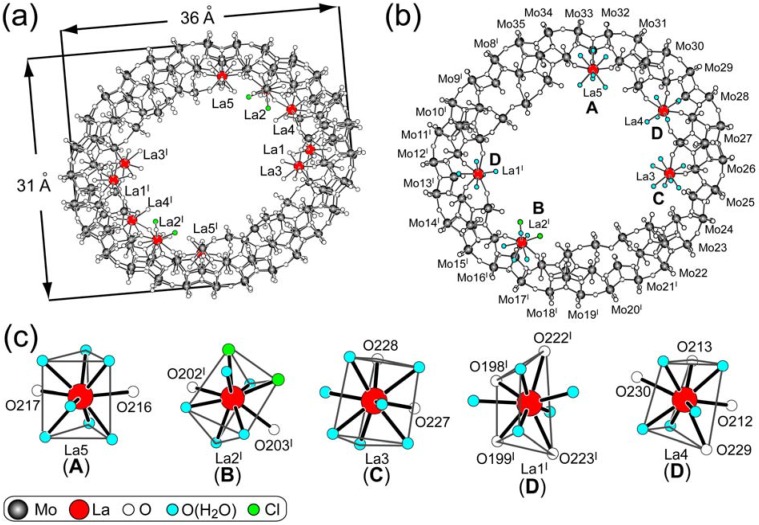
Structure of {Mo_134_La_10_} (a), four (A-D) modes for the coordination geometries of five La^3+^ ions at the half side of {Mo_134_La_10_}ring (b), and details of distorted TTP geometries for five La^3+^ ions in {Mo_134_La_10_} (c). Symmetry codes: I –x+2, -y+2, -z+3.

The *C_2h_*-symmetric 28-electron-reduced {Mo_142_(CH_3_CO_2_)_5_(C_2_H_5_CO_2_)} circular ring (with an outer-ring diameter of 34 Ǻ) reacts with La^3+^ in aqueous solutions at pH 3.5 (without any pH adjustment) to yield the elliptical ring of [Mo_134_O_416_H_20_(H_2_O)_46_{La(H_2_O)_5_}_4_{La(H_2_O)_7_}_4_{LaCl_2_(H_2_O)_5_}_2_]^10-^ ({Mo_134_La_10_}) with *C_i_* symmetry, isolated as the corresponding Na^+^ salt of Na_10_[Mo_134_O_416_H_20_(H_2_O)_46_{La(H_2_O)_5_}_4_{La(H_2_O)_7_}_4_{LaCl_2_(H_2_O)_5_}_2_]·144H_2_O. Figure 2a shows an elliptical *C_i_*-symmetric structure of {Mo_134_La_10_} and the long and short axes diameters for the outer ring of which are 36 and 31 Å, respectively. The manganometric redox titration analysis indicates the presence of 28 Mo^V^ centers in {Mo_134_La_10_}, indicating that 28 electrons injected in {Mo_142_(CH_3_CO_2_)_5_(C_2_H_5_CO_2_)} remain also in {Mo_134_La_10_} through the structural change from circular to elliptical ring. Interestingly, all the acetates and the propionate ligands coordinated in {Mo_142_(CH_3_CO_2_)_5_(C_2_H_5_CO_2_)} were removed through the coordination to ten La^3+^ ions within two inner rings (five for each) of {Mo_134_La_10_}.

No La^3+^ exists around an environment of {Mo_134_La_10_} in the lattice. All the La^3+^ sites of eight LaO_9_ (with La−O bond distances in 2.44(2)–2.71(2) Å) and two LaO_7_Cl_2_ (with La−Cl distances in 2.44(2)–2.71(2) Å) polyhedra in {Mo_134_La_10_} shows the distorted TTP geometries as well as other cases of elliptical {Mo_150_La_2_} and Japanese rice-ball {Mo_120_La_6_} species. However, one can remark four (A-D) modes of the TTP geometries within the inner ring with a resultant formation of *C_i_*-symmetric ring, different from the case (only D mode) for *C_2_*-symmetric {Mo_120_La_6_} and *C_2h_*-symmetric {Mo_150_La_2_}with the coordination of four O atoms belonging to MoO_6_ octahedra at two *head* and two *shoulder* Mo sites (as a 4-*fold* ligand for the ring) (Figure 1a) [8]. A-D modes of the TTP geometries in {Mo_134_La_10_} are depicted in Figure 2b where a half side of the {Mo_134_La_10_} ring is shown. A-mode indicates the LaO_2_(H_2_O)_7_ TTP geometry comprising two O atoms belonging to MoO_6_ octahedra at two *shoulder* sites, B-mode indicates the LaO_2_(H_2_O)_5_Cl_2_ TTP geometry with the same as A-mode but with the displacement of two aqua ligands with two Cl^-^, C-mode indicates the LaO_2_(H_2_O)_7_ TTP geometry comprising two O atoms belonging to the corner-sharing MoO_6_ octahedra at one *head* and one *shoulder* sites, and D-mode indicates the LaO_4_(H_2_O)_5_ TTP geometries of the same as for {Mo_150_La_2_} and {Mo_120_La_6_} (in Figure 1). All the 9-fold coordination geometries for A-D modes in the half side (Figure 2b) of the {Mo_134_La_10_} ring are schematically shown in Figure 2c, where each of the A-D modes indicates the distorted TTP geometry.

### 3.2. Calorimetry for the Coordination of {Mo_142_(CH_3_CO_2_)_5_(C_2_H_5_CO_2_)} to La^3+^

**Figure 3:**
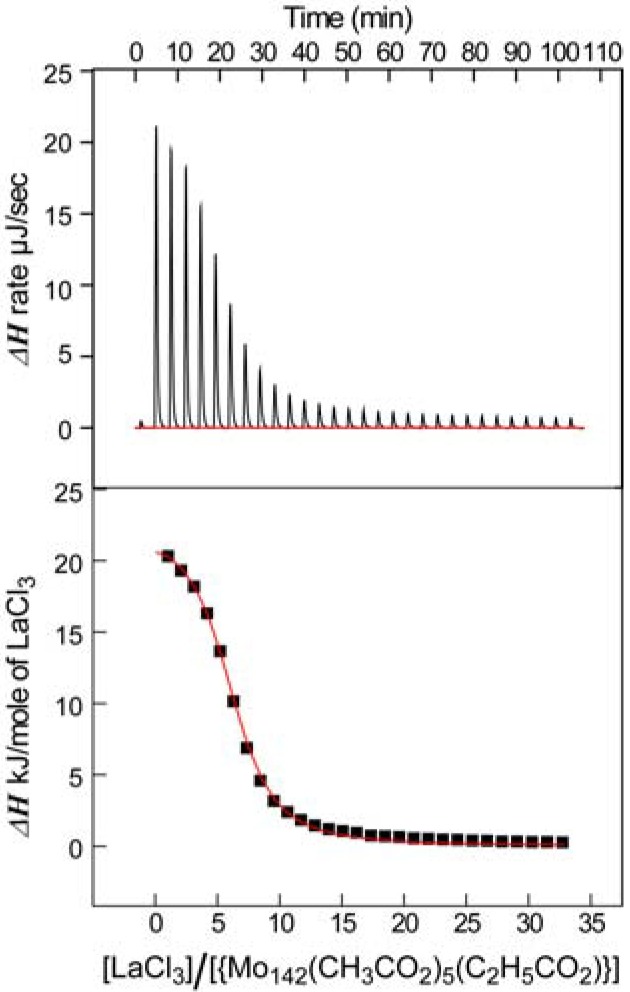
Binding isotherm for the titration of {Mo_142_(CH_3_CO_2_)_5_(C_2_H_5_CO_2_)} with LaCl_3_ at 293 K. An 20 μM solution of {Mo_142_(CH_3_CO_2_)_5_(C_2_H_5_CO_2_) was titrated with 10-μL 3 mM injections of LaCl_3_. Diagram of the enthalpy produced in each of injections of LaCl_3_ is shown in upper panel. The area under each injection signal was integrated and plotted against molar ratio in lower panel. The red-colored curve represents a nonlinear least squares fit of the reaction heat for the injection with the assumption of a single binding site.

The thermodynamic parameters for the La^3+^-induced change of the ring conformation of the {Mo_142_(CH_3_CO_2_)_5_(C_2_H_5_CO_2_)} circle to the {Mo_134_La_10_} ellipsoid are obtained by ITC technique. The ITC system let us directly measure the quantity of the heat evolved or absorbed in liquid samples on injecting reactants. The quantity of the evolved heat can be estimated by using the differential feedback power between the reference cell and the sample cell. An injection which results in the absorption of heat in the sample cell causes a positive change in the power, since the heat absorption requires feedback power. Thus, the La^3+^ titration of the aqueous solution of {Mo_142_(CH_3_CO_2_)_5_(C_2_H_5_CO_2_)} at pH 3.5 indicates the endothermic behavior which is deduced by plotting the integrated quantities of the reaction heat against the molar ratio of La^3+^ to {Mo_142_(CH_3_CO_2_)_5_(C_2_H_5_CO_2_)} and by applying a model based on a single set of identical sites. At 293 K, binding constant (*K*) = 9.9 × 10^4^ M^−1^, number of La^3+^ ions binding with a {Mo_142_(CH_3_CO_2_)_5_(C_2_H_5_CO_2_)} molecule (*n*) = 6.1, and molar enthalpy of La^3+^ ion (*ΔH*) = +22 kJ·mol^−1^ are estimated by using the best fit curve based on the model, as shown in Figure 3. The molar entropy of La^3+^ ion (*ΔS*) = +172 J·mol^−1^·K^−1^ is calculated from *K* and *ΔH* value (*ΔG* = −RTln*K* = *ΔH*−T*ΔS* ).

Table 1 lists the thermodynamic parameters obtained at various temperatures. At these temperatures, the coordination of {Mo_142_(CH_3_CO_2_)_5_(C_2_H_5_CO_2_)} to La^3+^ endothermically proceeded: with increasing temperature, the *K* value increases from 8.7 × 10^4^ M^−1^ at 278 K to 6.1 × 10^5^ M^−1^ at 333 K, in contrast to other parameters which indicate no significant change of *n ≈* 6 and *ΔH ≈* +22 kJ∙mol^-1^.

**Table 1:** Thermodynamic parameters obtained for the coordination of {Mo_142_(CH_3_CO_2_)_5_(C_2_H_5_CO_2_)} to La^3+^ ions at various temperatures.

**Temperature (K)**	278	293	313	333
***n***	5.6	6.1	6.2	5.8
**K (M^-1^)**	8.7 × 10^4^	9.9 × 10^4^	3.0 × 10^5^	6.1 × 10^5^
***ΔH* (kJ∙mol^-1^)**	19	22	22	23
***ΔS* (J·mol^−1^·K^−1^)**	164	172	175	182
***ΔG* (kJ∙mol^-1^)**	−28	−28	−33	−37

**Figure 4:**
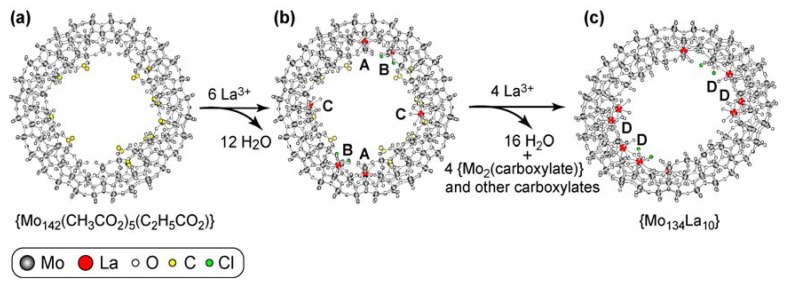
Two steps of the coordination of {Mo_142_(CH_3_CO_2_)_5_(C_2_H_5_CO_2_)} to La^3+^ in aqueous solutions. The removal of the coordinated carboxylates from the Mo-blue ring is governed by the kinetically controlled displacement of the carboxylates with aqua ligands. The La^3+^-coordination at six defect pockets (a→b) precedes the displacement of four {Mo_2_(carboxylate)} *linkers* (b→c): structure of {Mo_142_(CH_3_CO_2_)_5_(C_2_H_5_CO_2_)} ring (a); {Mo_134_(CH_3_CO_2_)_5_(C_2_H_5_CO_2_)(La)_6_} ring with two sets of A-C (one for each) modes (b); {Mo_134_La_10_} ring with additional two sets of two D modes (c).

Since for the ring modification of circle to ellipsoid the *ΔG* value (*ΔG* = *ΔH*−T*ΔS*) must be negative, the ring modification of {Mo_142_(CH_3_CO_2_)_5_(C_2_H_5_CO_2_)} to {Mo_134_La_10_} should occur through the sufficient entropy gain, which is an important factor for the ring modification from circle to ellipsoid. In the diluted aqueous solution of LaCl_3_ (<0.3 M), most of La^3+^ ions exist in the hydrated complex with {La(H_2_O)_9_}^3+^ nine-fold coordination [13]. In the {Mo_134_La_10_} formation system under the 1-mM concentration of LaCl_3_, thus, {La(H_2_O)_9_}^3+^ reacts with the {Mo_142_(CH_3_CO_2_)_5_(C_2_H_5_CO_2_)} ring to yield the A-D modes of the TTP geometries as revealed by the above structural analysis of {Mo_134_(La)_10_} through the displacement of two (for A-C modes) or four (for D mode) aqua ligands with *head* and *shoulder* MoO_6_ octahedral O atoms. Such a dehydratation process on the coordination to La^3+^ seems to provide a large entropy gain, if we consider that the coordination of {Mo_142_(CH_3_CO_2_)_5_(C_2_H_5_CO_2_)} to ten La^3+^ occurs through the liberation of twenty-eight aqua ligands. It is notable that the estimated binding number (*n*) of 6 implies that the reaction with a large heat change occurs under La^3+^/{Mo_142_(CH_3_CO_2_)_5_(C_2_H_5_CO_2_)} = 6:1 which corresponds to the number of defect pockets in {Mo_142_(CH_3_CO_2_)_5_(C_2_H_5_CO_2_)}. Thus, the ITC result for the La^3+^/ {Mo_142_(CH_3_CO_2_)_5_(C_2_H_5_CO_2_)} system strongly supports the preferential coordination of all the defect pockets of {Mo_142_(CH_3_CO_2_)_5_(C_2_H_5_CO_2_)} in two inner rings to six La^3+^ ions to yield a two sets of the A-C modes of the TTP geometries with the liberation of twelve aqua ligands, which follows the displacement of four {Mo_2_(carboxylate)} *linkers* with four La^3+^ to yield the D-mode of the TTP geometries with the liberation of sixteen aqua ligands. The occurrence of the displacement of the carboxylates-coordinated {Mo_2_} *linkers* with La^3+^ as a second step seems to be along with the fact that four {Mo_2_} *linkers* (two for each inner ring) still remain in {Mo_134_La_10_}. The removal of all the carboxylates coordinated in {Mo_142_(CH_3_CO_2_)_5_(C_2_H_5_CO_2_)} through the reaction with LaCl_3_ in the aqueous solutions is probably due to the kinetically controlled displacement of the carboxylates with aqua ligands. Figure 4 shows two steps for the coordinations of {Mo_142_(CH_3_CO_2_)_5_(C_2_H_5_CO_2_)} to ten La^3+^ ions which reveal the ring modification from circle to ellipsoid. The coordination of all the defect pockets to six La^3+^ ions (for A-C modes) with the liberation of twelve aqua ligands from [La(H_2_O)_9_]^3+^ results in a reduction (from 30- to 16-) of the negative charge of the anion ring, which seems to induce easier displacement of four positively (1+)-charged {Mo_2_(carboxylate)} *linkers* with four La^3+^ ions as well as an easy liberation (to more-positively (2+)-charged {Mo_2_}) of the carboxylates from four remaining {Mo_2_(carboxylate)} *linkers*.

### 3.3. ^139^La-NMR Spectrometry

**Figure 5:**
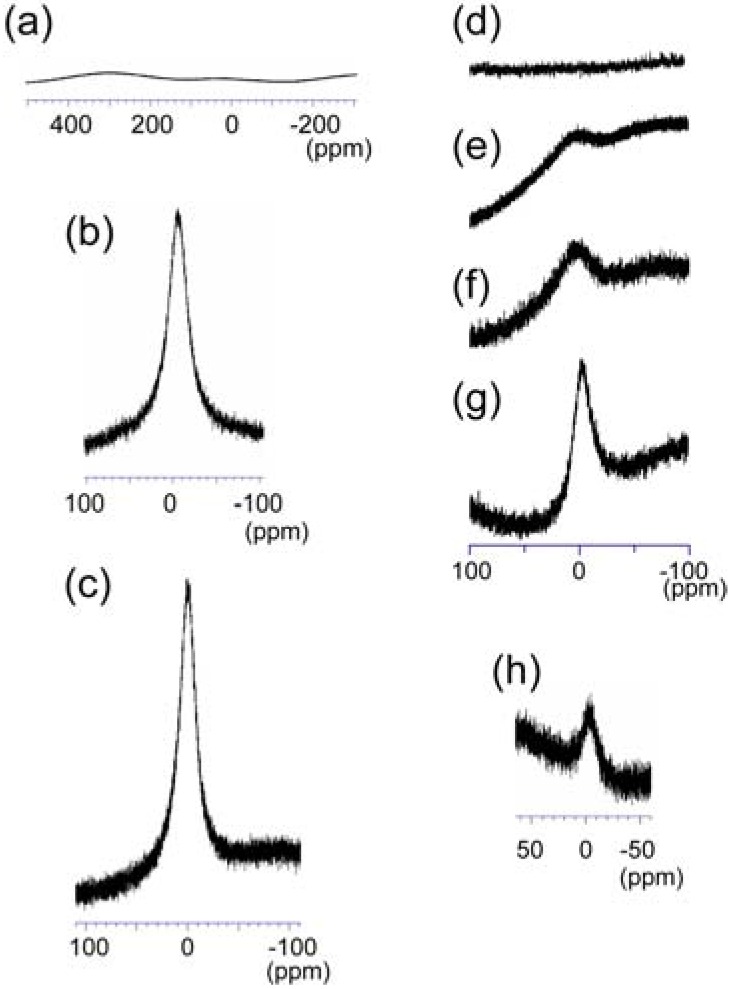
^139^La-NMR spectra: 0.5 mM {Mo_134_La_10_} (a); oxidization of (a) with HNO_3_ (b); 0.5 mM {Mo_134_La_10_} and 5 mM LaCl_3_ (c); 0.1 mM {Mo_142_(CH_3_CO_2_)_5_(C_2_H_5_CO_2_)} and 1 mM LaCl_3_ (d); and 15 mM LaCl_3_ (e); and 20 mM LaCl_3_ (f); and 50 mM LaCl_3_ (g); oxidization of the solution of (d) with HNO_3_ (h).

The ^139^La nuclide has high natural abundance (99.9 %) and is a very sensitive nuclide for NMR measurements. It has relatively high quadrupole moments: *I* = 7/2, *Q* = 0.22 × 10^−24^ cm^2^; therefore, the line width of the signal strongly depends on the site symmetry at La atoms. In our trial of ^139^La-NMR spectrometry of the {Mo_142_(CH_3_CO_2_)_5_(C_2_H_5_CO_2_)} coordination to La^3+^ the aqueous solution of {Mo_134_La_10_} showed no observable ^139^La−NMR signal. This arises from the inhomogeneous broadening due to the second order quadrupole interaction for the La^3+^ sites of the A-D modes in {Mo_134_La_10_}. Interestingly, the perfect oxidation of {Mo_134_La_10_} after adding a small amount of HNO_3_ (less than 10% v/v) gives rise to a signal around at −2.7 ppm which is assigned to the perfectly hydrated [La(H_2_O)_9_]^3+^ species formed by the oxidative decomposition of {Mo_134_La_10_}. Furthermore, the ^139^La-NMR spectrum of the [La^3+^]/[{Mo_134_La_10_}] = 10/1 system showed the broadened signal with ν_1/2_ = 703 Hz around at −0.3 ppm. Figure 5 shows ^139^La-NMR spectra of the aqueous solutions of {Mo_134_La_10_} (a), after oxidation (b), and the [La^3+^]/[{Mo_134_La_10_}] = 10/1 system (c). Together with the above readily coordination of {Mo_142_(CH_3_CO_2_)_5_(C_2_H_5_CO_2_)} to La^3+^ in aqueous solutions, the results in Figure 5a-c let us measure the ^139^La-NMR spectra of the aqueous solutions with the variety of [La^3+^]/[{Mo_142_(CH_3_CO_2_)_5_(C_2_H_5_CO_2_)}] ratio. Figure 5d-h show ^139^La-NMR spectra of [La^3+^]/[{Mo_142_(CH_3_CO_2_)_5_(C_2_H_5_CO_2_)}] = 10/1 (d), 15/1 (e), 20/1 (f), and 50/1(g). The [La^3+^]/[{Mo_142_(CH_3_CO_2_)_5_(C_2_H_5_CO_2_)}] = 10/1 system exhibits no signal, probably due to the perfect coordination of {Mo_142_(CH_3_CO_2_)_5_(C_2_H_5_CO_2_)} to ten La^3+^ ions to yield {Mo_134_La_10_} (Figure 5d). This can be also supported by the appearance of the peak around at −2.3 ppm when the [La^3+^]/[{Mo_142_(CH_3_CO_2_)_5_(C_2_H_5_CO_2_)}] = 10/1 system is perfectly oxidized by HNO_3_ (h). With increasing the ratio of La^3+^ a single broadened signal appears around at −0.6 ppm and its intensity increases with ν_1/2_ = 646 Hz for [La^3+^]/[{Mo_142_(CH_3_CO_2_)_5_(C_2_H_5_CO_2_)}] = 50/1. However, the intensities of broadened signals for [La^3+^]/[{Mo_142_(CH_3_CO_2_)_5_(C_2_H_5_CO_2_)}] = 15/1 and 20/1 are not sufficiently strong to estimate the linewidths.

## 4. Conclusions

The ring modification of the defect-containing *C_2h_*-{Mo_142_(CH_3_CO_2_)_5_(C_2_H_5_CO_2_)} circle to the *C_i_*-{Mo_134_La_10_} ellipsoid occurs through the coordination to La^3+^ in aqueous solutions. Together with the ITC calorimetry results, the structural comparison of two rings between {Mo_142_(CH_3_CO_2_)_5_(C_2_H_5_CO_2_)} and {Mo_134_La_10_} indicates that the coordination of the defect pockets to La^3+^ precedes the displacement of the carboxylates-coordinated {Mo_2_} *linkers* with La^3+^. Four modes (A-D) of LaO_9_/LaO_7_Cl_2_ -tricapped-trigonal-prismatic coordination (TTP) geometries (each one for A-C modes and two for D mode within each inner ring of {Mo_134_La_10_}) are revealed: A,{LaO_2_(H_2_O)_7_} (comprising the coordination of two O atoms at two *shoulder* MoO_6_ sites); B, {LaO_2_(H_2_O)_5_Cl_2_} (two O atoms at two *shoulder*); C, {LaO_2_(H_2_O)_7_} (two atoms at *head* and *shoulder*); and D, {LaO_4_(H_2_O)_5_} (four O atoms from two sets of *head* and *shoulder*). The coordination of all the defect pockets to six La^3+^ ions (for A-C modes) with the liberation of twelve aqua ligands from [La(H_2_O)_9_]^3+^ results in a reduction (from 30- to 16-) of the negative charge of the anion ring, which seems to induce easier displacement of positively-charged (carboxylate-coordinated){Mo_2_} *linkers* with La^3+^. The release of all the hydrophobic carboxylate ligands from the two inner rings on the coordination to La^3+^ ions indicates an increase of the hydrophilic La^3+^-TTP sites within the inner surface of the ring, suggesting the change of the ring surface from hydrophobic to hydrophilic property.

## Supporting Information

CIF data and checkCIF/PLATON report can be downloaded at http://www.mdpi.com/1996-1944/3/1/64/s1/.

Supplementary File 1

Supplementary File 2
