# Supplementary material for: Coordination of {Mo142} Ring to La3+ Provides Elliptical {Mo134La10} Ring with a Variety of Coordination Modes
Source: Materials (Basel). 2009 Dec 28;3(1):64–75. doi: 10.3390/ma3010064 (PMC5510172; doi:10.3390/ma3010064)
Supplement: Supplementary File 2 [file materials-03-00064-s002.pdf]

checkCIF/PLATON report (publication check)

No syntax errors found.
Please wait while processing ....

CIF dictionary
Interpreting this report

Datablock: _Mo134La

Bond precision: La- O = 0.0169 A Wavelength=0.71075

Cell: a=35.530(16) b=37.05(2) c=36.11(2)
alpha=90 beta=119.85(2) gamma=90

Temperature: 173 K

	Calculated	Reported
Volume	41228(38)	4123
Space group	P 21/c	P 1 21/c 1
Hall group	-P 2ybc	-P 2ybc
Moiety formula	Cl4 La10 Mo134 O520, 10(Na)	H516 CL4 LA10 MO134 NA10 O664
Sum formula	Cl4 La10 Mo134 Na10 O520	H516 CL4 LA10 MO134 NA10 O664
Mr	22936.76	25760.40
Dx,g cm-3	1.848	2.075
Z	2	2
Mu (mm-1)	2.555	2.574
F000	21072.0	24408.0
F000'	20616.65	
h,k,lmax	46,48,46	46,48,46
Nref	94596	94422
Tmin,Tmax	0.635,0.773	0.510,0.773
Tmin'	0.592	

Correction method= NUMERICAL

Data completeness= 0.998 Theta(max)= 27.490

R(reflections)= 0.0923(52411) wR2(reflections)= 0.2841(94422)

S = 1.012 Npar= 1940

The following ALERTS were generated. Each ALERT has the format
test-name_ALERT_alert-type_alert-level.
Click on the hyperlinks for more details of the test.

Alert level A

```
REFI014_ALERT_1_G  _refine_ls_hydrogen_treatment is missing
           Code for H-atom treatment.
           The following tests will not be performed
           HYDTR_01
GEOM006_ALERT_1_A  _geom_angle_atom_site_label_2 is missing
           Label identifying the atom site 2.
GEOM007_ALERT_1_A  _geom_angle_atom_site_label_3 is missing
           Label identifying the atom site 3.
CHEMW03_ALERT_2_A ALERT: The ratio of given/expected molecular weight as
           calculated from the _atom_site* data lies outside
           the range 0.90 <> 1.10
          From the CIF: _cell_formula_units_Z                   2
          From the CIF: _chemical_formula_weight          *******
          TEST: Calculate formula weight from _atom_site_*
          atom     mass    num     sum
          Mo       95.94  134.0012855.96
          O        16.00  520.00 8319.48
          La      138.91   10.00 1389.06
          Cl       35.45    4.00  141.81
          Na       22.99   10.00  229.90
          H         1.01    0.00    0.00
          Calculated formula weight           22936.21
PLAT220_ALERT_2_A Large Non-Solvent   Mo     Ueq(max)/Ueq(min) ...       7.22 Ratio
PLAT220_ALERT_2_A Large Non-Solvent    O     Ueq(max)/Ueq(min) ...      10.00 Ratio
PLAT242_ALERT_2_A Check Low        Ueq as Compared to Neighbors for        La1
PLAT242_ALERT_2_A Check Low        Ueq as Compared to Neighbors for       O195
PLAT242_ALERT_2_A Check Low        Ueq as Compared to Neighbors for       O206
PLAT242_ALERT_2_A Check Low        Ueq as Compared to Neighbors for       O225
PLAT602_ALERT_2_A VERY LARGE Solvent Accessible VOID(S) in Structure        !
PLAT043_ALERT_1_A Check Reported Molecular Weight ................   25760.40
PLAT044_ALERT_1_A Calculated and Reported Dx Differ ..............          ?
PLAT150_ALERT_1_A Volume as Calculated Differs from that Given ...    4123.00 Ang-3
```

Alert level B

```
CHEMS01_ALERT_1_B  The sum formula contains elements in the wrong order.
            H  precedes Cl
            Sequence must be alphabetical for inorganic structures.
DIFMN02_ALERT_2_B  The minimum difference density is < -0.1*ZMAX*1.00
            _refine_diff_density_min given =     -6.130
            Test value =     -5.700
DIFMX01_ALERT_2_B  The maximum difference density is > 0.1*ZMAX*1.00
            _refine_diff_density_max given =      8.970
            Test value =      5.700
PLAT097_ALERT_2_B Large Reported Max.  (Positive) Residual Density       8.97 eA-3
PLAT098_ALERT_2_B Large Reported Min.  (Negative) Residual Density      -6.13 eA-3
PLAT241_ALERT_2_B Check High      Ueq as Compared to Neighbors for       Mo67
PLAT242_ALERT_2_B Check Low       Ueq as Compared to Neighbors for        La3
PLAT242_ALERT_2_B Check Low       Ueq as Compared to Neighbors for       Mo60
PLAT242_ALERT_2_B Check Low       Ueq as Compared to Neighbors for       Mo63
PLAT242_ALERT_2_B Check Low       Ueq as Compared to Neighbors for       O220
```

Alert level C

```
DIFMN03_ALERT_1_C  The minimum difference density is < -0.1*ZMAX*0.75
            The relevant atom site should be identified.
DIFMX02_ALERT_1_C  The maximum difference density is > 0.1*ZMAX*0.75
            The relevant atom site should be identified.
RFACR01_ALERT_3_C  The value of the weighted R factor is > 0.25
            Weighted R factor given   0.284
PLAT084_ALERT_2_C High R2 Value .................................       0.28
PLAT165_ALERT_3_C Nr. of Status R Flagged Non-Hydrogen Atoms .....        18
PLAT241_ALERT_2_C Check High      Ueq as Compared to Neighbors for       Mo45
PLAT241_ALERT_2_C Check High      Ueq as Compared to Neighbors for       Mo57
PLAT241_ALERT_2_C Check High      Ueq as Compared to Neighbors for       Mo61
PLAT241_ALERT_2_C Check High      Ueq as Compared to Neighbors for       Mo62
PLAT242_ALERT_2_C Check Low       Ueq as Compared to Neighbors for        La2
PLAT242_ALERT_2_C Check Low       Ueq as Compared to Neighbors for        La4
PLAT242_ALERT_2_C Check Low       Ueq as Compared to Neighbors for        La5
PLAT242_ALERT_2_C Check Low       Ueq as Compared to Neighbors for       O174
PLAT242_ALERT_2_C Check Low       Ueq as Compared to Neighbors for       O202
PLAT242_ALERT_2_C Check Low       Ueq as Compared to Neighbors for       O208
PLAT242_ALERT_2_C Check Low       Ueq as Compared to Neighbors for       O231
PLAT041_ALERT_1_C Calc. and Reported SumFormula    Strings  Differ          ?
PLAT042_ALERT_1_C Calc. and Reported MoietyFormula Strings  Differ          ?
PLAT068_ALERT_1_C Reported F000 Differs from Calcd (or Missing)...          ?
PLAT151_ALERT_1_C No su (esd) Given on Volume ....................          ?
PLAT161_ALERT_4_C Missing or Zero su (esd) on x-coordinate for ...       MO57
PLAT161_ALERT_4_C Missing or Zero su (esd) on x-coordinate for ...       MO61
PLAT161_ALERT_4_C Missing or Zero su (esd) on x-coordinate for ...       MO62
PLAT161_ALERT_4_C Missing or Zero su (esd) on x-coordinate for ...       MO67
PLAT161_ALERT_4_C Missing or Zero su (esd) on x-coordinate for ...        O72
PLAT161_ALERT_4_C Missing or Zero su (esd) on x-coordinate for ...        O73
PLAT161_ALERT_4_C Missing or Zero su (esd) on x-coordinate for ...        O74
PLAT161_ALERT_4_C Missing or Zero su (esd) on x-coordinate for ...        O75
PLAT161_ALERT_4_C Missing or Zero su (esd) on x-coordinate for ...        O84
PLAT161_ALERT_4_C Missing or Zero su (esd) on x-coordinate for ...        O85
PLAT161_ALERT_4_C Missing or Zero su (esd) on x-coordinate for ...        O86
PLAT161_ALERT_4_C Missing or Zero su (esd) on x-coordinate for ...        O87
PLAT161_ALERT_4_C Missing or Zero su (esd) on x-coordinate for ...        O88
PLAT161_ALERT_4_C Missing or Zero su (esd) on x-coordinate for ...        O89
PLAT161_ALERT_4_C Missing or Zero su (esd) on x-coordinate for ...        O90
PLAT161_ALERT_4_C Missing or Zero su (esd) on x-coordinate for ...       O102
PLAT161_ALERT_4_C Missing or Zero su (esd) on x-coordinate for ...       O103
PLAT161_ALERT_4_C Missing or Zero su (esd) on x-coordinate for ...       O104
PLAT162_ALERT_4_C Missing or Zero su (esd) on y-coordinate for ...       MO57
PLAT162_ALERT_4_C Missing or Zero su (esd) on y-coordinate for ...       MO61
PLAT162_ALERT_4_C Missing or Zero su (esd) on y-coordinate for ...       MO62
PLAT162_ALERT_4_C Missing or Zero su (esd) on y-coordinate for ...       MO67
PLAT162_ALERT_4_C Missing or Zero su (esd) on y-coordinate for ...        O72
PLAT162_ALERT_4_C Missing or Zero su (esd) on y-coordinate for ...        O73
PLAT162_ALERT_4_C Missing or Zero su (esd) on y-coordinate for ...        O74
PLAT162_ALERT_4_C Missing or Zero su (esd) on y-coordinate for ...        O75
PLAT162_ALERT_4_C Missing or Zero su (esd) on y-coordinate for ...        O84
PLAT162_ALERT_4_C Missing or Zero su (esd) on y-coordinate for ...        O85
PLAT162_ALERT_4_C Missing or Zero su (esd) on y-coordinate for ...        O86
PLAT162_ALERT_4_C Missing or Zero su (esd) on y-coordinate for ...        O87
PLAT162_ALERT_4_C Missing or Zero su (esd) on y-coordinate for ...        O88
PLAT162_ALERT_4_C Missing or Zero su (esd) on y-coordinate for ...        O89
PLAT162_ALERT_4_C Missing or Zero su (esd) on y-coordinate for ...        O90
PLAT162_ALERT_4_C Missing or Zero su (esd) on y-coordinate for ...       O102
PLAT162_ALERT_4_C Missing or Zero su (esd) on y-coordinate for ...       O103
PLAT162_ALERT_4_C Missing or Zero su (esd) on y-coordinate for ...       O104
PLAT163_ALERT_4_C Missing or Zero su (esd) on z-coordinate for ...       MO57
PLAT163_ALERT_4_C Missing or Zero su (esd) on z-coordinate for ...       MO61
```

```
PLAT163_ALERT_4_C Missing or Zero su (esd) on z-coordinate for ...      MO62
PLAT163_ALERT_4_C Missing or Zero su (esd) on z-coordinate for ...      MO67
PLAT163_ALERT_4_C Missing or Zero su (esd) on z-coordinate for ...       O72
PLAT163_ALERT_4_C Missing or Zero su (esd) on z-coordinate for ...       O73
PLAT163_ALERT_4_C Missing or Zero su (esd) on z-coordinate for ...       O74
PLAT163_ALERT_4_C Missing or Zero su (esd) on z-coordinate for ...       O75
PLAT163_ALERT_4_C Missing or Zero su (esd) on z-coordinate for ...       O84
PLAT163_ALERT_4_C Missing or Zero su (esd) on z-coordinate for ...       O85
PLAT163_ALERT_4_C Missing or Zero su (esd) on z-coordinate for ...       O86
PLAT163_ALERT_4_C Missing or Zero su (esd) on z-coordinate for ...       O87
PLAT163_ALERT_4_C Missing or Zero su (esd) on z-coordinate for ...       O88
PLAT163_ALERT_4_C Missing or Zero su (esd) on z-coordinate for ...       O89
PLAT163_ALERT_4_C Missing or Zero su (esd) on z-coordinate for ...       O90
PLAT163_ALERT_4_C Missing or Zero su (esd) on z-coordinate for ...      O102
PLAT163_ALERT_4_C Missing or Zero su (esd) on z-coordinate for ...      O103
PLAT163_ALERT_4_C Missing or Zero su (esd) on z-coordinate for ...      O104
PLAT711_ALERT_1_C BOND    Unknown or Inconsistent Label ..........      O328
                    NA(5)  O(328)
PLAT751_ALERT_4_C Bond    Calc     2.18487, Rep   2.184(14) ......  Senseless su
              MO(57-O(195   1.555   1.555
PLAT751_ALERT_4_C Bond    Calc     2.18730, Rep     2.19(2) ......  Senseless su
              MO(57-O(221   1.555   1.555
PLAT751_ALERT_4_C Bond    Calc     2.17832, Rep   2.178(15) ......  Senseless su
              MO(61-O(206   1.555   1.555
PLAT751_ALERT_4_C Bond    Calc     2.03553, Rep   2.035(19) ......  Senseless su
              MO(61-O(224   1.555   1.555
PLAT751_ALERT_4_C Bond    Calc     2.16309, Rep   2.163(16) ......  Senseless su
              MO(62-O(225   1.555   1.555
PLAT751_ALERT_4_C Bond    Calc     2.00670, Rep     2.01(2) ......  Senseless su
              MO(62-O(226   1.555   1.555
PLAT751_ALERT_4_C Bond    Calc     2.28485, Rep     2.28(2) ......  Senseless su
              MO(67-O(231   1.555   1.555
```

Alert level G

```
FORMU01_ALERT_2_G  There is a discrepancy between the atom counts in the
            _chemical_formula_sum and the formula from the _atom_site* data.
            Atom count from _chemical_formula_sum:H516 Cl4 La10 Mo134 Na10 O664
            Atom count from the _atom_site data:  Cl4 La10 Mo134 Na10 O520
CELLZ01_ALERT_1_G Difference between formula and atom_site contents detected.
CELLZ01_ALERT_1_G ALERT: Large difference may be due to a
            symmetry error - see SYMMG tests
           From the CIF: _cell_formula_units_Z     2
           From the CIF: _chemical_formula_sum  H516 Cl4 La10 Mo134 Na10 O664
           TEST: Compare cell contents of formula and atom_site data

           atom    Z*formula  cif sites diff
           H       1032.00      0.00 1032.00
           Cl         8.00      8.00    0.00
           La        20.00     20.00    0.00
           Mo       268.00    268.00    0.00
           Na        20.00     20.00    0.00
           O       1328.00   1040.00  288.00
PLAT072_ALERT_2_G SHELXL First Parameter in WGHT Unusually Large..      0.16
```

```
  13 ALERT level A = In general: serious problem
  10 ALERT level B = Potentially serious problem
  82 ALERT level C = Check and explain
   5 ALERT level G = General alerts; check

  16 ALERT type 1 CIF construction/syntax error, inconsistent or missing data
  31 ALERT type 2 Indicator that the structure model may be wrong or deficient
   2 ALERT type 3 Indicator that the structure quality may be low
  61 ALERT type 4 Improvement, methodology, query or suggestion
   0 ALERT type 5 Informative message, check
```

---

checkCIF publication errors

Alert level A

```
PUBL002_ALERT_1_A  The contact author's address is missing,
            _publ_contact_author_address.
PUBL012_ALERT_1_A  _publ_section_abstract is missing.
            Abstract of paper in English.
```

Alert level G

```
PUBL013_ALERT_1_G The _publ_section_comment (discussion of study) is
```

```
           missing. This is required for a full paper submission (but is
           optional for an electronic paper).
PUBL017_ALERT_1_G The _publ_section_references section is missing or
           empty.
```

```
   2 ALERT level A = Data missing that is essential or data in wrong format
   2 ALERT level G = General alerts. Data that may be required is missing
```

Publication of your CIF

You should always attempt to resolve as many as possible of the alerts in all categories. Often the minor alerts point to easily fixed oversights, errors and omissions in your CIF or refinement strategy, so attention to these fine details can be worthwhile. In order to resolve some of the more serious problems it may be necessary to carry out additional measurements or structure refinements. However, the nature of your study may justify the reported deviations from the submission requirements of the journal and these should be commented upon in the discussion or experimental section of a paper - after all, they might represent an interesting feature.

If level A alerts remain, which you believe to be justified deviations, and you intend to submit this CIF for publication in Acta Crystallographica Section C or Section E, you should additionally insert an explanation in your CIF using the Validation Reply Form (VRF) below. Your explanation will be considered as part of the review process.

If you intend to submit to another section of Acta Crystallographica or Journal of Applied Crystallography or Journal of Synchrotron Radiation, you should make sure that at least a basic structural check is run on the final version of your CIF prior to submission.

```
# start Validation Reply Form
_vrf_PUBL002_GLOBAL
;
PROBLEM: The contact author's address is missing,
RESPONSE: ...
;
_vrf_PUBL012_GLOBAL
;
PROBLEM: _publ_section_abstract is missing.
RESPONSE: ...
;
_vrf_GEOM006__Mo134La
;
PROBLEM: _geom_angle_atom_site_label_2 is missing
RESPONSE: ...
;
_vrf_GEOM007__Mo134La
;
PROBLEM: _geom_angle_atom_site_label_3 is missing
RESPONSE: ...
;
_vrf_CHEMW03__Mo134La
;
PROBLEM: ALERT: The ratio of given/expected molecular weight as
RESPONSE: ...
;
_vrf_PLAT220__Mo134La
;
PROBLEM: Large Non-Solvent   Mo     Ueq(max)/Ueq(min) ...       7.22 Ratio
RESPONSE: ...
;
_vrf_PLAT242__Mo134La
;
PROBLEM: Check Low       Ueq as Compared to Neighbors for        La1
RESPONSE: ...
;
_vrf_PLAT602__Mo134La
;
PROBLEM: VERY LARGE Solvent Accessible VOID(S) in Structure        !
RESPONSE: ...
;
_vrf_PLAT043__Mo134La
;
PROBLEM: Check Reported Molecular Weight ................   25760.40
RESPONSE: ...
;
_vrf_PLAT044__Mo134La
```

```
;
PROBLEM: Calculated and Reported Dx Differ .............          ?
RESPONSE: ...
;
_vrf_PLAT150__Mo134La
;
PROBLEM: Volume as Calculated Differs from that Given ...    4123.00 Ang-3
RESPONSE: ...
;
# end Validation Reply Form
```

If you wish to submit your CIF for publication in Acta Crystallographica Section C or E, you should upload your CIF via the web. If your CIF is to form part of a submission to another IUCr journal, you will be asked, either during electronic submission or by the Co-editor handling your paper, to upload your CIF via our web site.

PLATON version of 13/08/2009; check.def file version of 12/08/2009

Datablock _Mo134La - ellipsoid plot

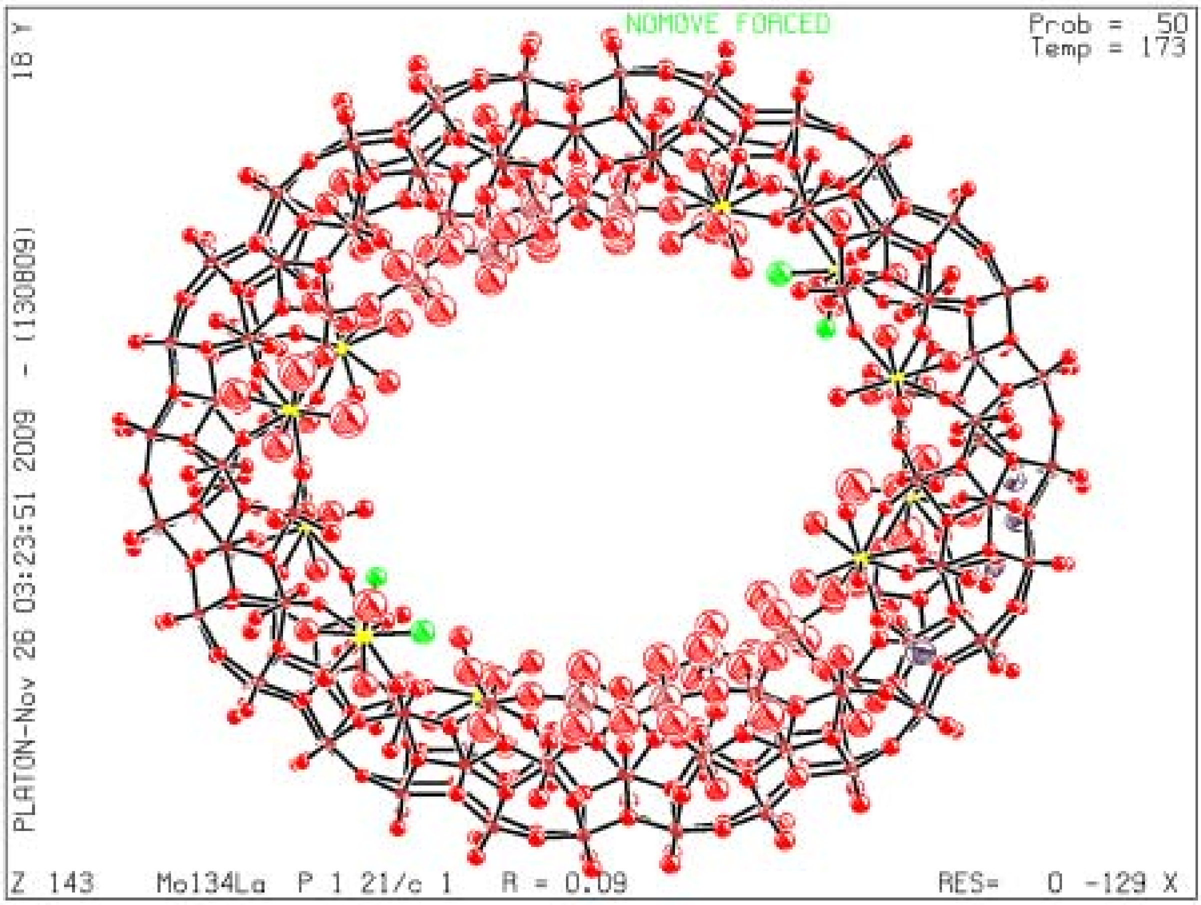

Download CIF editor (publCIF) from the IUCr
Download CIF editor (enCIFer) from the CCDC
Test a new CIF entry
